# MYL5 as a Novel Prognostic Marker is Associated with Immune Infiltrating in Breast Cancer: A Preliminary Study

**DOI:** 10.1155/2023/9508632

**Published:** 2023-02-17

**Authors:** Minghe Lv

**Affiliations:** ^1^Department of Radiation Oncology, Fudan University Shanghai Cancer Center, Fudan University, Shanghai, China; ^2^Department of Oncology, Shanghai Medical College, Fudan University, Shanghai, China; ^3^Shanghai Clinical Research Center for Radiotherapy Oncology, Shanghai Key Laboratory of Radiation Oncology, Shanghai 200032, China

## Abstract

**Background:**

Myosin light chain plays a vital regulatory function in a large-scale cellular physiological procedure, however, the role of myosin light chain 5 (MYL5) in breast cancer has not been reported. In this study, we aimed to elucidate the effects of MYL5 on clinical prognosis and immune cell infiltration, and further explore the potential mechanism in breast cancer patients.

**Methods:**

In this study, we first explored the expression pattern and prognostic value of MYL5 in breast cancer across multiple databases, including Oncomine, TCGA, GTEx, GEPIA2, PrognoScan, and Kaplan–Meier Plotter. The correlations of MYL5 expression with immune cell infiltration and associational gene markers in breast cancer were analyzed by using the TIMER, TIMER2.0, and TISIDB databases. The enrichment and prognosis analysis of MYL5-related genes were implemented by using LinkOmics datasets.

**Results:**

We found that there was a low expression of MYL5 in breast cancer than in corresponding normal tissue by analyzing the data from Oncomine and TCGA datasets. Furthermore, research showed the prognosis of the MYL5 high-expression group was better than the low-expression group in breast cancer patients. Furthermore, MYL5 expression is markedly related to the tumor-infiltrating immune cells (TIICs), including cancer-associated fibroblast, B cell, CD8^+^ T cell, CD4^+^ T cell, macrophage, neutrophil, and dendritic cell, and related to immune molecules as well as the associated gene markers of TIICs.

**Conclusion:**

MYL5 can serve as a prognostic signature in breast cancer and is associated with immune infiltration. This study first offers a relatively comprehensive understanding of the oncogenic roles of MYL5 for breast cancer.

## 1. Introduction

Breast cancer, the most common malignancy in females, is a leading cause of cancer-related incidence and mortality around the world [1–3]. Breast invasive carcinoma (BRCA), the most common type of breast cancer, is generally poorly differentiated and has a poorer prognosis than the other types, and is the most common type of breast cancer, accounting for approximately 80 percent of breast cancer [4]. With advances in early diagnosis and treatment, many patients have been successfully treated, with an average 5-year survival rate of about 90% [5]. However, about 20% to 25% of patients are diagnosed with locally advanced breast cancer, and early recurrence and death are the main causes of therapeutic failure in these patients [6]. Therefore, to further demonstrate the molecular mechanism of mammary carcinoma, it is pressing to probe new therapeutic methods.

Myosins consist of two heavy chains, two nonphosphorylable base light chains, and two phosphorylable regulatory light chains. Myosins have been shown to be involved in cell contraction, cell signaling, endocytosis, vesicle transport, and protein/RNA localization [7, 8], and are the actin-dependent molecular motor that uses the energy hydrolyzed by ATP to move along actin filaments and generate force, which plays a key role in regulating tumor progression and metastasis [9–11]. The changes in myosins expression could be used to predict therapeutic outcomes and, in some cases, provide attractive targets for the development of antitumor drugs [12]. Myosin light chain 5 (MYL5) encodes one of the light chains of myosin, which is a component of the hexameric ATPase cellular motor protein myosin. However, to date, there have been few clinical studies to indicate the clinical value and functional role of MYL5 in tumors, especially breast cancer. Therefore, in view of the complexity of tumor occurrence and progression in BRCA (breast invasive carcinoma), it is of great importance for us to clarify the correlation between MYL5 and clinical prognosis, as well as the potential molecular mechanism of great significance in breast cancer.

In this study, we attempted to explore the effect of MYL5 expression on the prognosis of patients with pan-cancer through bioinformatics analysis using public data sets, and further explore the potential molecular mechanism of MYL5 on the clinical prognosis of breast cancer.

## 2. Methods and Materials

### 2.1. Gene Expression Analysis

We used the TIMER2.0 (Tumor Immune Estimation Resource, Version 2) and GEPIA2 (Gene Expression Profiling Interactive Analysis, Version 2) tools to gain the MYL5 expression difference between pan-cancer and normal tissues. By using the GEPIA2, we analyzed the correlation between the expression of MYL5 and the pathological stages of tumors, and results were exhibited by using the box or violin figure. The data of these results in this study from the Oncomine database (https://www.oncomine.org) were obtained before its discontinuation.

### 2.2. Survival Prognosis Analysis

We employed the “Survival Map” module of GEPIA2 to explore the effects of MYL5 expression on the OS (Overall survival) and DFS (Disease-free survival) across all cancers in the TCGA dataset. We also used the data from PrognoScan (https://dna00.bio.kyutech.ac.jp/PrognoScan/index.html) and Kaplan–Meier plotter (https://kmplot.com/analysis/) databases to further analyze the effects of the expression of MYL5 on outcome in cancers. The effect of both MYL5 and clinicopathological factors on patient prognosis in breast tumor patients was analyzed by the Kaplan–Meier plotter tool.

### 2.3. Immune Infiltrating Analysis and Prognosis Analysis

We used the TIMER2 web server to gain the relationship between the expression of MYL5 and cancer-associated fibroblasts across all TCGA tumors. The EPIC, MCPCOUNTER, XCELL, and TIDE algorithms were applied for immune infiltration estimations. The *P* values and partial correlation (cor) values were obtained via the purity-adjusted Spearman's rank correlation test. The data were displayed by a heatmap and a scatter plot. Additionally, the relationship between MYL5 expression and other immune infiltration cells was determined by using the TIMER (https://cistrome.org/TIMER/) databases. The relationship between the gene markers of TIICs and the expression of MYL5 were analyzed by GEPIA2 and TIMER2.0 tools.

### 2.4. TISIDB Database Analysis

TISIDB database (https://cis.hku.hk/TISIDB) is a portal for tumor and immune system interactions that integrates multiple heterogeneous data types. We used the data from the TISIDB dataset to analyze the association between MYL5 expression and lymphocytes, immune modulators (including immunosuppressants and immunostimulants), and chemokines.

### 2.5. LinkedOmics Database Analysis

We employed the LinkedOmics database (https://www.linkedomics.org/login.php) to explore 32 TCGA cancer-associated multidimensional databases. The differentially expressed genes correlated with MYL5 were screened from the TCGA BRCA queue by the LinkFinder module, and the association of Pearson correlation coefficient test results was displayed in the volcano map and heat map, respectively. Function module analysis of Gene Ontology biological process (GO_BP), Gene Ontology molecular function (GO_MF), Gene Ontology cellular component (GO_CC), and Kyoto Encyclopedia of Genes and Genomes (KEGG) pathways by the Gene Set Enrichment Analysis (GSEA) in the link interpreter module.

### 2.6. Statistical Analysis

The data from the Oncomine database were presented as *p* values determined in *t*-tests, fold changes, and gene ranks. In their respective analyses, survival maps were generated using the PrognoScan, Kaplan–Meier Plotter, TIMER, TIMER2.0, and GEPIA2 databases, including HR and *p* values or *p* values from log-rank tests. Spearman's and Pearson's correlation analyses were used to measure the degree of correlation between specific variables. *p* < 0.05 was considered statistically significant, if not specially noted.

## 3. Results

### 3.1. The Different Expression of MYL5 Gene between Pan-Cancer and Normal Tissue

**Figure 1:**
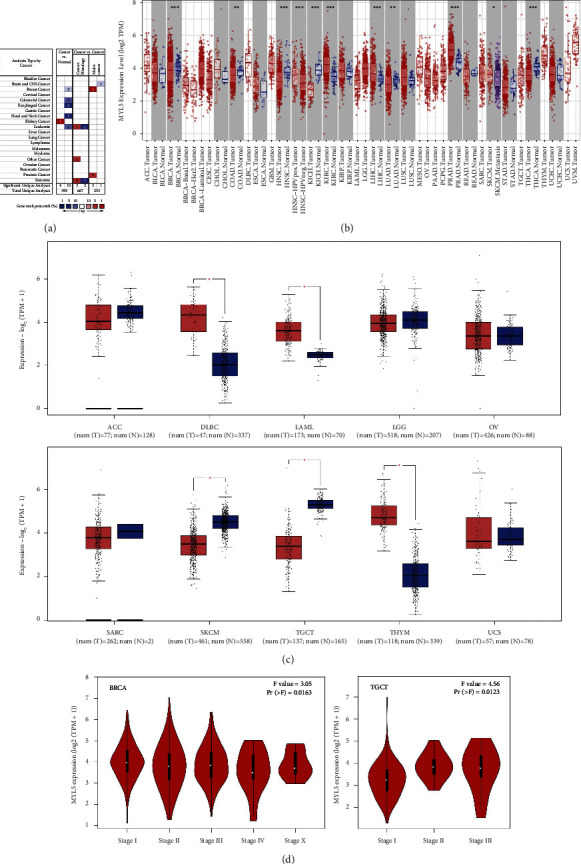
The expression levels of the MYL5 gene in pan-tumors and normal tissues. The expression levels of MYL5 in different types of tumor tissues and normal tissues were analyzed by using the Oncomine database (the threshold of *p* value is 0.01, fold change is 2, and gene ranking is all) (a), TIMER 2.0 (b) and GEPIA2 (c) tools (d). The expression levels of the MYL5 gene were analyzed by the main pathological stages of BRCA and TGCT. Log2 (TPM + 1) was applied for the log-scale. ^*∗*^*p* < 0.05, ^*∗∗*^*p* < 0.01, and ^*∗∗∗*^*p* < 0.001.

In this study, we first used the data of the Oncomine database to analyze the difference in MYL5 gene expression, and the results showed that compared with corresponding normal tissues, the expression of MYL5 was decreased in breast cancer, colorectal cancer, esophageal cancer, gastric cancer, head and neck cancer, and leukemia, but was elevated in kidney cancer (Figure 1(a)). The results that were analyzed by TIMER 2.0 tool showed that the expression of the MYL5 gene was significantly elevated in kidney renal clear cell carcinoma (KIRC, *p* < 0.001), liver hepatocellular carcinoma (LIHC, *p* < 0.001), and prostate adenocarcinoma (PRAD, *p* < 0.001), compared to the expression of MYL5 gene of normal tissues but was markedly decreased in breast invasive carcinoma (BRCA, *p* < 0.001), colon adenocarcinoma (COAD, *p* < 0.01), head and neck squamous cell carcinoma (HNSC, *p* < 0.001), kidney Chromophobe (KICH, *p* < 0.01), lung adenocarcinoma (LUAD, *p* < 0.01), and thyroid carcinoma (THCA, *p* < 0.001) (Figure 1(b)). Because there were no matched normal tissues for adrenocortical carcinoma (ACC), lymphoid neoplasm diffuse large B-cell lymphoma (DLBC), acute myeloid leukemia (LAML), brain lower grade glioma (LGG), ovarian serous cystadenocarcinoma (OV), sarcoma (SARC), skin cutaneous melanoma (SKCM), testicular germ cell tumors (TGCG), thymoma (THYM), and uterine carcinosarcoma (UCS), we further investigated the differential expression of MYL5 between tumors and normal tissues by using the GEPIA 2 tools to match TCGA normal and GTEx data. As shown in Figure 1(c), there was a higher expression of MYL5 in DLBC, LAML, and THYM than in corresponding control tissues, but a lower expression of MYL5 in SKCM and TGCT. Furthermore, we employed the GEPIA 2.0 tool to analyze the relationship between MYL5 expression and clinical stage, and the results showed that the expression of MYL5 in BRCA and TGCT correlated obviously with the clinical stage (Figure 1(d)). The data of this part indicated that MYL5 expression existed a significant difference between pan-cancer and normal tissue, which deserved further investigation.

### 3.2. Survival Analysis of MYL5 Expression by GEPIA 2 Tool

**Figure 2:**
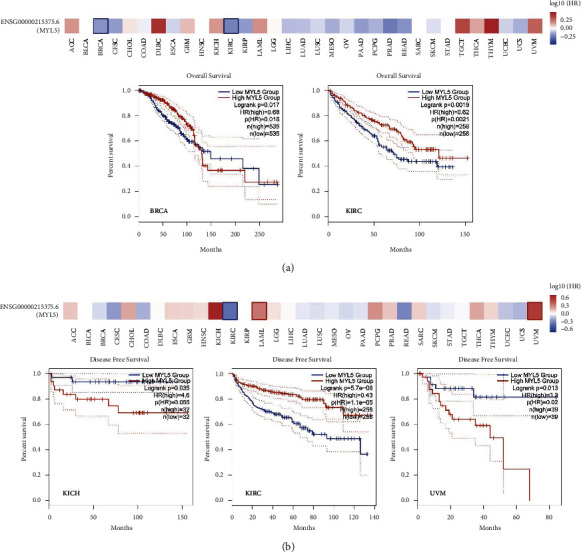
The prognosis analysis of MYL5 expression in the TCGA dataset. The effects of MYL5 expression on overall survival (a) and disease free survival (b) in different tumors were analyzed by using the GEPIA 2 tool. The survival map and Kaplan–Meier curves with positive results were displayed.

To study the effect of the MYL5 gene on survival, we used the GEPIA 2 tool to analyze the data divided into high-expression and low-expression groups from TCGA and GEO databases, and the heatmap and Kaplan–Meier plot of overall survival (OS) and disease-free survival (DFS) were displayed (Figures 2(a) and 2(b)). We found that in BRCA patients, there was significantly longer overall survival (OS) in the high-expression of MYL5 group than in the low-expression group (HR = 0.68, *p* (HR) = 0.018, and long-rank*p*=0.017), as well as in KIRC patients (HR = 0.62, *p* (HR) = 0.0021, and long-rank*p*=0.0019). In addition, the disease free survival (DFS) of KIRC patients in the MYL5 high-expression group was longer than in the MYL5 low-expression group (HR = 0.43, *p* (HR) = 0.000011, and long-rank*p*=0.0000057); however, the DFS of KICH (HR = 4.6, *p* (HR) = 0.055, and long-rank*p*=0.035) and UVM (HR = 3.3, *p* (HR) = 0.02, and long-rank*p*=0.013) patients in MYL5 high-expression group was lower than in MYL5 low-expression group. All data manifested that the MYL5 gene could be a potential and novel prognosis factor and it could be of benefit to clinical diagnosis and therapy for different cancers.

### 3.3. Survival Analysis Data of MYL5 in Kaplan–Meier Plotter and PrognoScan Databases

**Figure 3:**
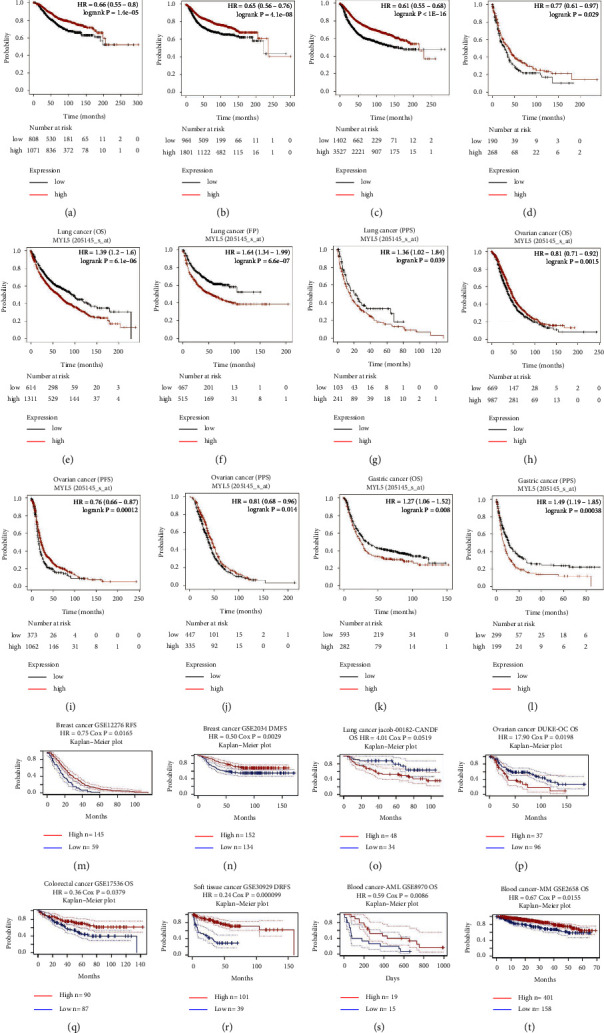
The prognosis analysis of MYL5 expression in Kaplan–Meier plotter and PrognoScan datasets. Correlation between MYL5 expression and prognosis of various types of cancer in Kaplan–Meier (a–l) and PrognoScan datasets (m–t). OS: overall survival, PFS: progression-free survival, PPS: postprogression survival, FP: first progression, DRFS: distant recurrence free survival, DMFS: distant metastases-free survival, RFS: relapse-free survival, AML: acute myelocytic leukemia, and MM: multiple myeloma.

To verify the effect of MYL5 on prognosis in pan-cancer, we further explored the prognosis difference between the MYL5 high-expression group and MYL5 low-expression group by Kaplan–Meier plotter and PrognoScan databases. The results showed that in Kaplan–Meier plotter and PrognoScan databases, the overall survival (OS), distant metastases-free survival (DMFS), relapse-free survival (RFS), and postprogression survival (PPS) of breast cancer patients in the MYL5 high-expression group were all significantly longer than the MYL5 low-expression group (Figures 3(a)–3(d)). However, in lung cancer, we found that the OS, first progression (FP), and PPS in MYL5 high-expression group were markedly shorter than the MYL5 low-expression group (Figures 3(e)–3(g)). The OS, progression-free survival (PFS), and PPS of ovarian cancer patients in the MYL5 high-expression group were obviously longer than the MYL5 low-expression group (Figures 3(h)–3(j)). As for gastric cancer patients, the results, as shown in Figures 3(k) and 3(l), displayed that the OS and PPS in the MYL5 high-expression group were shorter than the MYL5 low-expression group. All data from the Kaplan–Meier plotter dataset of this part demonstrated that MYL5 could be a potential and poor prognostic factor for lung cancer and gastric cancer patients, but a better prognostic biomarker for breast cancer and ovarian cancer patients. To further verify the conclusion among the previous data about the effect of the MYL5 gene on prognosis, we used the data from the PrognoScan dataset to study whether MYL5 expression had contributed to a better prognosis for special types of cancers. As shown in Figures 3(m) and 3(n), the RFS and DMFS of breast cancer patients in the MYL5 high-expression group were significantly longer than the MYL5 low-expression group, which kept in with the survival data from the Kaplan–Meier plotter dataset and these data further indicated that MYL5 served as a potential and favorable biomarker on diagnosis in breast cancer. Similarly, as for lung cancer, the result of OS in the PrognoScan dataset supported the conclusion that the OS of lung cancer patients in the MYL5 high-expression expression group was shorter than the MYL5 low-expression group, indicating that MYL5 could be a poor prognostic factor for lung cancer (Figure 3(o)). However, in the ovarian cancer survival data from the PrognoScan dataset (*n* = 1656), we found that the OS in MYL5 high-expression group was shorter than the MYL5 low-expression group, which contained to the analysis of survival data from the Kaplan–Meier plotter dataset (*n* = 123) (Figure 3(p)), and we speculated that the contradictory results could be caused by the number of samples. In addition, we also found the prognostic difference between the MYL5 high-expression group and the low-expression group among colorectal cancer, soft tissue cancer, acute myelocytic leukemia (AML), and multiple myeloma (MM). Results showed that the OS of colorectal cancer, AML, and MM patients and the distant recurrence-free survival (DRFS) of soft tissue cancer in the MYL5 high-expression group were significantly longer than the MYL5 low-expression group (Figures 3(q)–3(t)). Therefore, all data of this part demonstrated that MYL5 might be a potential and novel biomarker for specific cancer patients' clinical diagnosis and therapy.

### 3.4. The Effect of Different Clinicopathological Factors on the Expression of MYL5 Gene and Clinical Prognosis in Breast Cancer

In previous results, we found that MYL5 expression was linked with great breast cancer patient prognosis in Kaplan–Meier plotter and PrognoScan datasets. So, in this part, we first investigated the correlation of MYL5 expression with clinicopathological factors in breast cancer by analyzing the data from the TCGA dataset. The results showed that MYL5 expression significantly correlated with T stage, pathologic stage, histological type, race, PR status, ER status, HER2 status, and molecular subtype (Table 1). To further investigate the effect of MYL5 on survival prognosis in breast cancer, we continued to employ the Kaplan–Meier plotter to analyze the effect of different clinicopathological factors on the expression of the MYL5 gene and clinical prognosis. As shown in Table 2, we found that in ER-negative or HER2-negative breast cancer patients, high-expression MYL5 was conducive to prolong the overall survival (OS) and relapse-free survival (RFS), and, in ER-positive or HER2-positive breast cancer patients, there was only longer RFS in MYL5 high-expression group than the low-expression group. In addition, the RFS of the high-expression of the MYL5 was longer than the low-expression group in PR-negative breast cancer. We divided breast cancer patients into different intrinsic subtypes, including basal (triple-negative), luminal A, luminal B, and HER2^+^, and explore the role of MYL5 on prognosis in each subtype. The results showed that MYL5 expression could prolong the OS in basal and HER2^+^ breast cancer patients, and increase the RFS among basal, luminal A, and HER2^+^ breast cancer patients. For lymph node-negative patients, MYL5 expression significantly lengthened the OS and RFS in breast cancer, and for lymph node-positive patients, MYL5 expression only prolonged the RFS. We also found that the grade of breast cancer markedly affected the role of MYL5 expression on the RFS. For TP53 status, while type breast cancer patients' OS and RFS were elevated in MYL5 high-expression group. The data of this part demonstrated the stratification analysis about the value of MYL5 expression on survival prognosis, providing evidence for the value of MYL5 to apply diagnosis and therapy on different clinical types of breast cancer.

### 3.5. Correlation Analysis between MYL5 Expression and Cancer-Associated Fibroblast Infiltration

**Figure 4:**
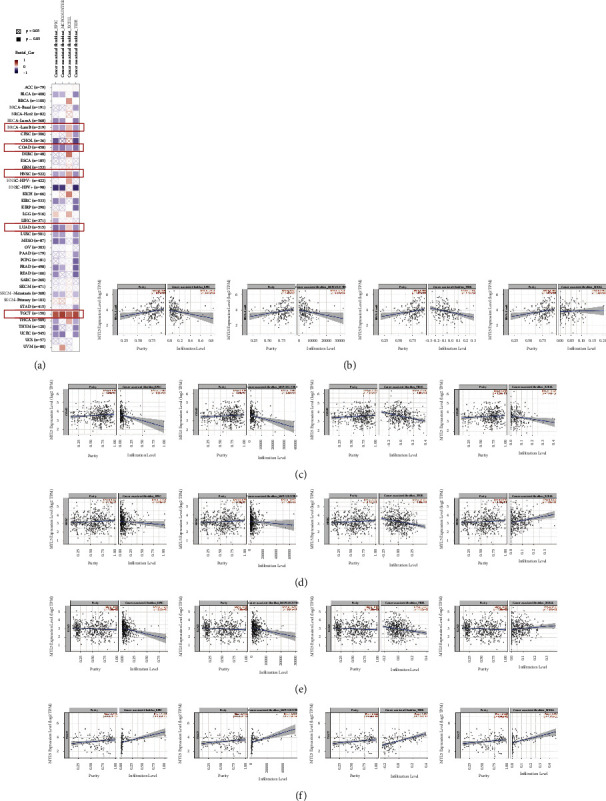
Correlation analysis between MYL5 expression and immune infiltration of cancer associated fibroblasts by using the TIMER2.0 tool. Different algorithms were used to explore the potential correlation between the expression level of the MYL5 gene and the infiltration level of cancer-associated fibroblasts across all types of cancer in TCGA. The heatmap (a), and the scatter plot of BRCA-lumB (b), COAD (c), HNSC (d), LUAD (e), and TGCT (f) in four datasets were given. *p* value was analyzed by the Spearman's correlation.

After conducting a prognosis analysis, we first further investigated the relationship of MYL5 expression with cancer-associated fibroblasts (CAF). The correlation of MYL5 expression with CAF infiltrate in pan-cancer was exhibited by the heatmap through employing the TIMER2.0 tool, with the EPIC, MCPCOUNTER, XCELL, and TIDE algorithms, respectively (Figure 4(a)). Then, the scatter diagrams showed the detailed contact between the two. In BRCA-LumB, HNSC, and LUAD, the MYL5 expression was significantly and negatively correlated to the CAF infiltrate by using EPIC, MCPCOUNTER, and TIDE algorithms, but was positively related to the CAF infiltrate via XCELL algorithm (Figures 4(b), 4(d), and 4(e)). Interestingly, as shown in Figure 4(c), we found that in COAD, the MYL expression obviously and negatively correlated with the CAF infiltrate through the four algorithms; however, in TGCT, the expression of MYL5 markedly and positively related to the infiltrate of CAF via the four algorithms (Figure 4(f)).

### 3.6. Association between MYL5 Expression and Immnue Infiltrate Cells in Different Molecular Subtypes of BRCA

**Figure 5:**
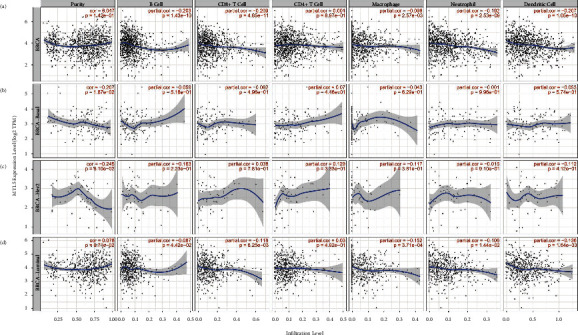
Association between MYL5 expression and immune infiltrate cells in different molecular subtypes of BRCA. We used the TIMER tool to analyze the correlation of MYL5 expression with B cell, M1 macrophage, M2 macrophage, tumor-associated macrophage (TAM), monocytes, and T cell exhaustion among BRCA (a), BRCA-Basal (b), BRCA-Her2 (c), and BRCA-luminal (d).

In this study, we further investigated the correlation of MYL5 expression with B cell, CD8^+^ T cell, CD4^+^ T cell, macrophage, neutrophil, and dendritic cell (DC). The results showed that in all BRCA patients, these immune infiltrate cells distinctly correlated with the expression of MYL5 (Figure 5(a)). When distinguishing the molecular subtype, we found that only in BRCA-Luminal, the expression of MYL5 related to the infiltration of immune cells, including B cell, CD8^+^ T cell, macrophage, neutrophil, and DC, which suggested that the correlation between MYL5 expression and infiltrating immune cells in BRCA was mainly reflected by the luminal classification of BRCA (Figures 5(b)–5(d)).

### 3.7. Prognostic Analysis of MYL5 Expression and Immune Infiltrate Cells in Different Molecular Subtypes of BRCA

**Figure 6:**
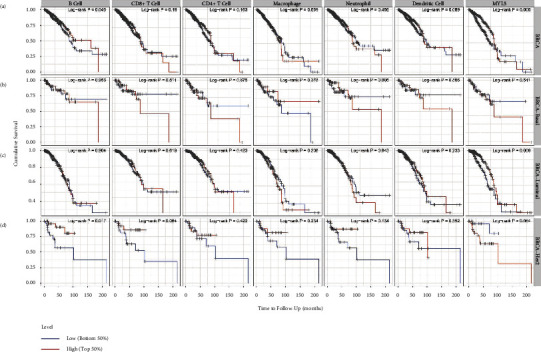
Prognostic analysis of MYL5 expression and immune infiltrate cells among different molecular subtypes of BRCA. We used the TIMER tool to analyze the correlation of MYL5 with B cell, M1 macrophage, M2 macrophage, tumor-associated macrophage (TAM), monocytes, and T cell exhaustion among BRCA (a), BRCA-Basal (b), BRCA-luminal (c), and BRCA-Her2 (d).

In this part, we studied the effect of MYL5 expression and immune infiltrate cells, including B cell, CD8^+^T cell, CD4^+^T cell, macrophage, neutrophil, and dendritic cell (DC) on prognosis in BRCA, BRCA-Basal, BRCA-Her2, and BCRA-Luminal, respectively. In BRCA patients who were not divided into different molecular subtypes, we found that MYL5 expression and B cell infiltrate collectively affected the prognosis for breast cancer patients (Figure 6(a)). If distinguishing molecular subtypes in BRCA, we detected that in BRCA-Basal, all indexes did not exert an impact on the patient's prognosis; however, in BRCA-Luminal, low-expression of MYL5 indicated a poor prognosis, and in BRCA-Her2, the high-level infiltrate of B cell displayed a great prognosis (Figures 6(b)–6(d)). The analysis of these data via employing the TIMER tool suggested that MYL5 might be great prognostic biomarkers for BRCA-Luminal patients.

### 3.8. Correlation Analysis of MYL5 with Immnue Cell Marks in BRCA

**Figure 7:**
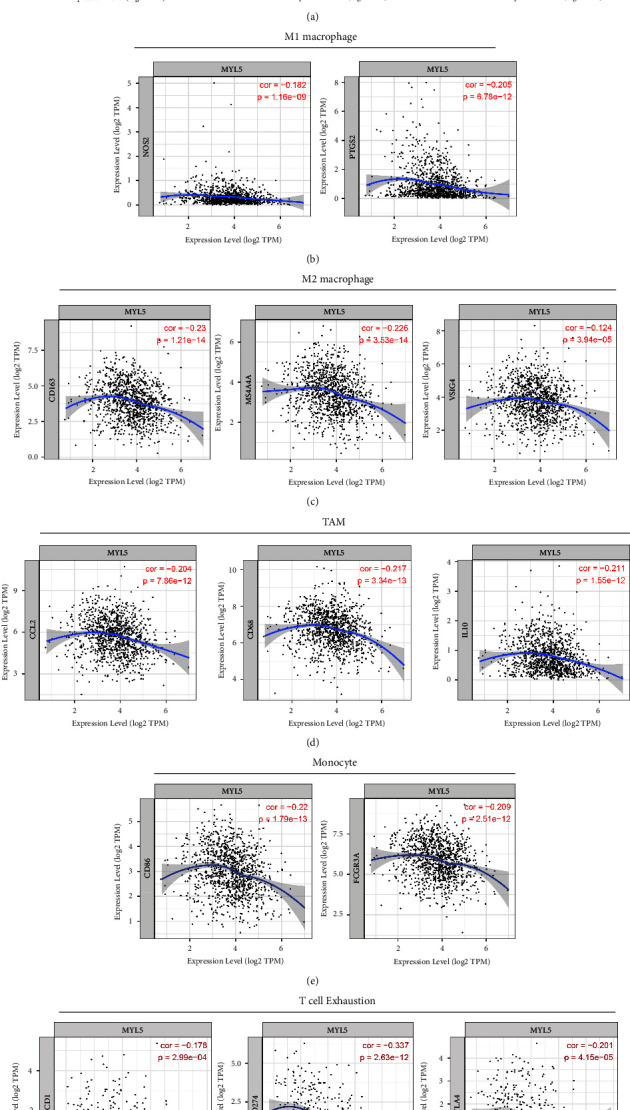
Correlation analysis between MYL5 expression and immunological marker set via using the TIMER tool in BRCA. Scatterplots of correlations between MYL5 expression and gene markers of B cell (a), M1 macrophage (b), M2 macrophage (c), tumor-associated macrophage (TAM) (d), monocytes (e), and T cell exhaustion (f) were displayed in breast cancer, without purity adjustment.

To further investigate the potential relationship between MYL5 expression and TIICs in BRCA, we explored the correlations between MYL5 and several immune cell markers, including B cell, M1/M2 macrophage, tumor-associated macrophage (TAM), monocyte, and T cell exhaustion, by TIMER and GEPIA2. As shown in Figure 7(a), MYL5 expression significantly correlated with B cell markers, inculding KRT20 (CD20), CD19, and CD38. As for M1 macrophage markers, NOS2 and PTGS2 (COX) also obviously correlated with the expression of MYL5 (Figure 7(b)). CD163, MS4A4A, and VSIG4 served as the associational markers of M2 macrophage and were correlated with MYL5 expression (Figure 7(c)). The gene markers of TAM, including CCL2, CD68, and IL-10, are markedly related to the expression of the MYL5 gene in BRCA (Figure 7(d)). We found that CD86 and CD16 (FCGR3A) as gene markers for monocyte were significantly and negatively relevant to MYL5 expression (Figure 7(e)). Especially, the associational markers of T cell exhaustion including PD1, PD-L1, and CTLA4 were deemed to correlate with tumor immune escape, and the results exhibited that the expression of MYL5 negatively and significantly correlated with them, which additionally indicated that MYL5 could serve as a good prognostic biomarker for BRCA patients. To verify the conclusion about the association between the expression of MYL5 and immune cell markers, we employed the GEPIA2 tool to analyze the relationship of MYL5 with B cell, M1/M2 macrophage, TAM, monocyte, and T cell exhaustion in breast cancer and normal tissue, severally. As shown in Table 3, the results of the correlation between MYL5 expression and gene markers of B cell, M1/M2 macrophage, TAM, monocyte, and T cell exhaustion brought into correspondence with the previous result in Figure 7, showing that these markers all negatively and significantly correlated with MYL5 expression in BRCA. Particularly, we found that NOS2, CCL2, CD68, CD86, CD16 (FCGR3A), and PD-L1 (CD274) also have a significant correlation with the expression of MYL5. The data of this part demonstrated that MYL5, a novel, and prognostic biomarker, signally correlated with the immune cell infiltration and their correlative gene markers.

### 3.9. Relation between MYL5 with Immune Molecules

**Figure 8:**
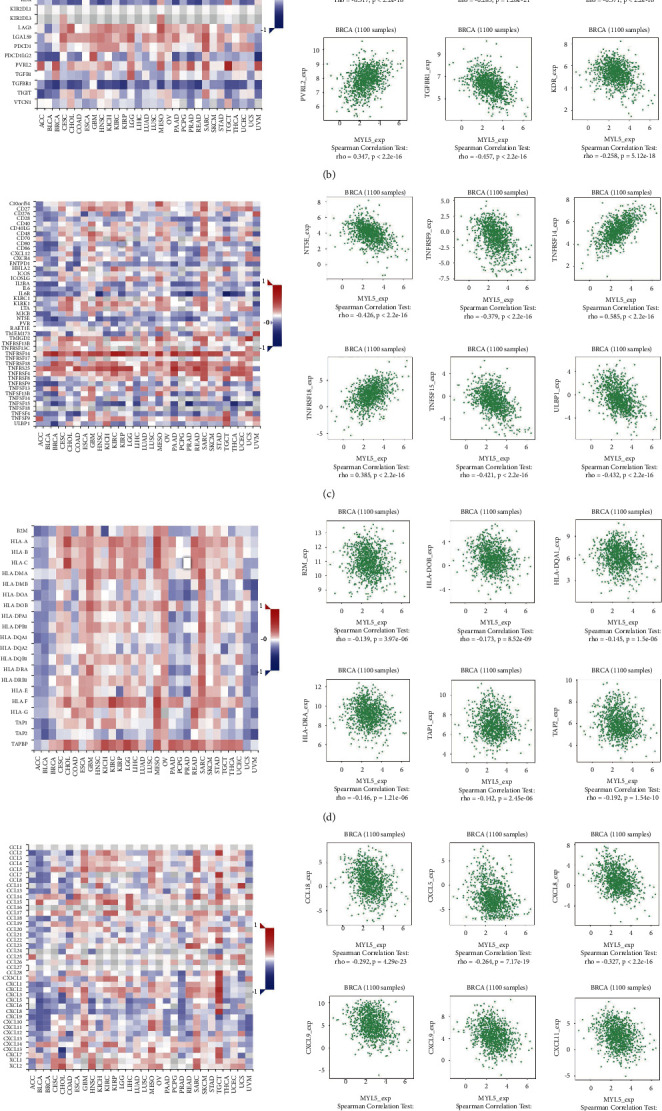
Connections of the expression level of MYL5 of BRCA patients with lymphocytes, immunomodulators, and chemokines in the TISIDB database. (a) The relationship between TILs and MYL5. (b–d) The relations between immunomodulators and MYL5. (e) and (f) Correlations of chemokines (or receptors) and MYL5.

To further improve the cognition of the relationship between MYL5 expression and immune infiltration, we studied the associations between the expression of the MYL5 gene and various immune markers, which included the 28 TIL types of immune-related signatures, three kinds of immunomodulators, chemokines, and receptors. Correlations between the expression of MYL5 and various immune markers in BRCA were obtained from the TISIDB database. As shown in Figure 8(a), the heatmap displayed the correlations between MYL5 and tumor-infiltrating lymphocytes (TILs) in pan-cancer, and the scatter plots of the top 6 of the absolute value of *p* in BRCA were given. Immunomodulators can be further divided into three groups including immune inhibitor, immunostimulator, and major histocompatibility complex (MHC) molecules. Figures 8(b)–8(d) respectively showed the connection of MYL5 expression with immune inhibitor, immunostimulator, and MHC molecules, and the heatmap in pan-cancer and the top 6 scatter plots of the absolute *p* values in BRCA were displayed. The relationship between MYL5 expression and chemokines in pan-cancer was presented by heatmap, and especially, the top 6 scatter plots of the absolute *p* values showed the negative correlation of the two in BRCA (Figure 8(e)). Similarly, in Figure 8(f), the association between MYL5 expression and receptors in pan-cancer was also presented by heatmap, and the top 6 scatter plots of the absolute *p* values showed the correlation of the two in BRCA. Therefore, it was confirmed that MYL5 participated widely in modulating various immune molecules in BRCA to affect immune infiltration in the tumor microenvironment.

### 3.10. Coexpression Network of MYL5 Gene in Breast Cancer

**Figure 9:**
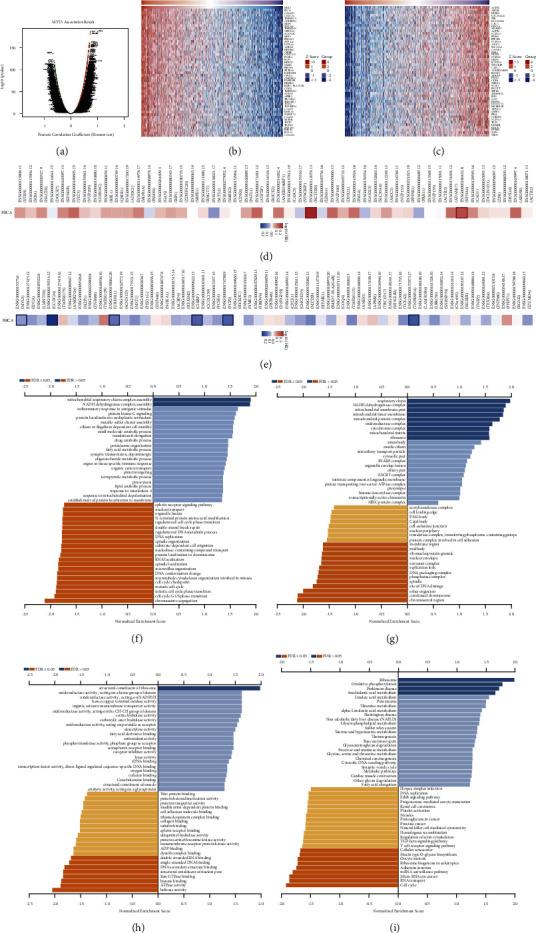
The coexpression genes with MYL5 of BRCA patients in the LinkedOmics database. (a) The whole obviously correlated genes with MYL5 distinguished by the Pearson test in BRCA. (b) and (c) Top 50 genes positively and negatively correlated with MYL5 in BRCA were respectively displayed by the heatmap. (d) and (e) Survival map of the top 50 genes negatively and positively associated with MYL5 in BRCA. (f–i) GO analysis (biological process), GO analysis (molecular function), GO analysis (cellular component), and KEGG pathways of MYL5 in BRCA cohort.

To further investigate the biological function of MYL5 in breast cancer, we used the data from the LinkedOmics dataset to explore the coexpression pattern of MYL5 in TCGA-BRCA. As shown in Figure 9(a), it displayed that 7785 genes (dark red dots) positively correlated with MYL5, and 7613 genes (dark green dots) negatively correlated with MYL5 (Figures 9(b) and 9(c)). What is noteworthy is that as shown in Figure 9(d), the top 48 (two genes (LOC284441 and KIAA0754) were not found in GEPIA2) negatively genes with a highly owned probability of becoming high-risk markers in BRCA, of which 36/48 genes had a disadvantageous hazard ratio (HR), especially CDK8 and NUP205. In contrast, there were 33 of the top 50 genes with protective HR in the top 50 positively significant genes, mainly including CCDC24, UBXN11, PCP2, and TNFRSF14 (Figure 9(e)).

The results of GO analysis (Biological Process) showed that coexpressed genes of MYL5 mainly participated in mitochondrial respiratory chain complex assembly, NADH dehydrogenase complex assembly, chromosome segregation, cell cycle G1/S phase transition, etc. (Figure 9(f)). As shown in Figure 9(g), GO analysis (molecular function) displayed that MYL5 mainly joined structural constituent of ribosome, helicase activity, ATPase activity, histone binding, etc. Coexpressed genes of MYL5 primarily took part in the respiratory chain, NADH dehydrogenase complex, mitochondrial membrane part, chromosomal, etc. via GO analysis (cellular component) (Figure 9(h)). The bar chart of KEGG pathways analysis from the LinkedOmics database revealed that coexpression genes of MYL5 are mainly involved in ribosome, oxidative phosphorylation, arachidonic acid metabolism, cell cycle, etc. mRNA surveillance pathway might be mainly involved in the effect of coexpression genes of MYL5 on breast cancer pathogenesis (Figure 9(i)).

The data of this part indicated a wide influence of the MYL5 expression network on the prognosis, proliferation, and metabolism of BRCA.

## 4. Discussion

Recent studies showed that myosins play vital roles in the physiological or pathological processes of cells, which included cytokinesis failure, chromosomal [13] and centrosomal amplification [14], multipolar spindle formation, and DNA microsatellite instability [10]. These progresses were all preconditions of cancer formation and development. Furthermore, myosins activated many processes of malignancy invasion and metastasis, mainly including cell migration, adhesion, protrusion formation, cycle arrest, and apoptosis inhibition [15]. Recently, studies have incrementally indicated that the myosin superfamily played an important role during oncogenesis and tumor-related diseases [16–18]. For example, Myosin light chain (MLC) kinase inhibitors could block the invasion and adhesion of human pancreatic tumor cell lines [19]. The upregulation of myosin VA by Snail was involved in tumor cell migration and metastasis [20]. As the research progresses, the presence of myosin II, such as MYL1, MYL2, and MYL9 in the nuclei of several cell types, and their transcriptional function is gradually reported [21, 22]. However, little was known about the existence and tumorigenic role of MYL5 in many types of tumors. In a previous study, we used the expression of WDR6 (WD repeat domain 6) between pan-cancer and normal tissue by employing the bioinformation analysis and exploring the immunological and prognostic role in lung cancer patients [23]. Similarly, in this study, we first analyzed the expression of the MYL5 gene between pan-cancer and normal tissues, finding that by analyzing the data of the Oncomine database, compared with corresponding normal tissues, MYL5 were underexpressed in breast cancer, colorectal cancer, esophageal cancer, gastric cancer, head and neck cancer, and leukemia, but were highly expressed in kidney cancer. Meanwhile, our results from the TCGA database showed that the expression of the MYL5 gene was significantly elevated in KIRC, LIHC, and PRAD, compared to the expression of the MYL5 gene in normal tissues, but was markedly decreased in BRCA, COAD, HNSC, KICH, LUAD, and THCA. Therefore, the expression difference of MYL5 between cancer tissues and normal tissues was worth further investigation, in order to reveal the value of MYL5 in clinical diagnosis and therapy of tumor patients.

Myosins played a vital function not only in tumorigenesis but also could be used as an impressible signature for cancer diagnosis [24]. Studies showed that MYO5B (myosin VB) may become an important biomarker for gastric cancer because the expression of MYO5B was downregulated in gastric cancer and the inactivation of MYO5B may contribute to tumorigenesis [25]. Myosin VI (MYO6), serving as a sensitive biomarker, was highly expressed in the Golgi apparatus of prostate cancer cells [26]. In the present study, in order to elucidate the potential value of MYL5 on breast cancer, we investigated the effects of MYL5 expression on survival prognosis, mainly including OS, RFS, DMFS, PFS, FP, and PPS. The results showed that in Kaplan–Meier plotter and PrognoScan databases, the OS, DMFS, RFS, and PPS of breast cancer patients were significantly prolonged in the MYL5 high-expression group, compared with the MYL5 low-expression group. However, we found that in lung cancer, the OS, FP, and PPS in MYL5 high-expression group were markedly shorter than the MYL5 low-expression group. The OS, progression-free survival (PFS), and PPS in the MYL5 high-expression group for ovarian cancer patients were obviously longer than in the MYL5 low-expression group. As for gastric cancer patients, the OS and PPS in the MYL5 high-expression group were shorter than the MYL5 low-expression group. All data from the Kaplan–Meier plotter dataset of this part demonstrated that MYL5 could be a potential and poor prognostic factor for lung cancer and gastric cancer patients, but a better prognostic biomarker for breast cancer and ovarian cancer patients. To further verify the conclusion among the previous data about the effect of the MYL5 gene on prognosis, we used the data from the PrognoScan dataset to study whether MYL5 expression had contributed to a better prognosis for special types of cancers. All data analysis from the PrognoScan database showed results that were mainly consistent with the Kaplan–Meier database, except for ovarian cancer. We speculated that the contradictory survival analysis results between the two databases for ovarian cancer could be mainly caused by the number of samples. This was an interesting phenomenon, which was worthy of further molecular experiments and animal models to verify its accuracy in the future. Therefore, all data of this part demonstrated that MYL5 might be a potential and novel biomarker for specific cancer patients' clinical diagnosis and therapy.

Tumor cells exist in a complex tumor microenvironment (TME) [27]. It is all known that tumor-infiltrating immune cells, as prominent components of the tumor microenvironment, were closely linked to the initiation, progression, or metastasis of neoplasm [28, 29]. In most cases, the primary role of TME exerted immunosuppression, which blocked anticancer immunity and sustain tumor progression [30]. The immunosuppressive effect of TME was regulated by all immune cell types with immunomodulatory activities [27]. Studies showed that macrophages situated in TME tend to become tumor-associated macrophages (TAMs) to drive tumor progression, invasion, and metastasis [31]. In our study, we found that with the analysis data via using TIMER and GEPIA tool, MYL5 expression negatively correlated with macrophages infiltration in breast tumor patients, and negatively and markedly correlated with the gene markers of macrophages and TAMs, indicating that MYL5 might affect the prognosis via regulating the TAMs in TME, thereby causing in longer survival in breast tumor patients. In addition, deeper and more research indicated that tumor-infiltrating dendritic cells (DC) are inclined to promote immunosuppression and tolerance in TME, rather than drive anticancer immunity [32]; Our results showed that the expression of the MYL5 gene was negatively relevant to DC infiltration in breast cancer, and this could suggest that MYL5 gene could affect the prognosis by regulating DC infiltration level. Neutrophils which were recruited into cancer, are usually polarized towards the N2-subtype with protumor roles [32]. Our study found that MYL5 expression also correlated with neutrophils. T cells are the main expression cells of anticancer response. Most tumors reduced T cell-mediated immune response in various ways, including inhibiting T cell transport to the tumor, interfering with antigen-presenting cells, and effector T cells [30, 33]. Tumor cells expressed PD-L1 (programmed cell death protein ligand 1) or PD-L2 (programmed cell death protein ligand 2) ligands that match the T-cell PD-1 protein, preventing them from finding the tumor and sending signals to the immune system to attack the tumor, directly leading to T-cell failure [34]. Clinically, blocking this event, namely the application of anti-PD-1 and anti-PD-L1 antibodies, could not only promote the proliferation of T cells but also restore the cytotoxic response of T cells to tumor cells [30, 34]. In the present study, MYL5 expression negatively correlated with the markers of T cells exhaustion, such as PDCD1 (PD-1), CD274 (PD-L1), and CTLA4 (cytotoxic T-lymphocyte antigen 4), which indicated that MYL5 could play a positive role in prolonging survival prognosis for breast cancer patients. The LinkedOmics database analysis further showed that MYL5 not only has an important influence on the prognosis of BRCA patients but also most of the genes coexpressed with MYL5 in BRCA are positively or negatively correlated with the prognosis of BRCA patients. In addition, these coexpressed genes were significantly focused on ATP-related and metabolism-related pathways, which coincided with the known function of MYL5. All signs indicate that MYL5 can play a vital role in affecting the prognosis of BRCA.

Our previous data manifested that the low-expression level of MYL5 was associated with poor prognosis, and further suggested that MYL5 could serve as a good prognostic biomarker to diagnose and treat breast cancer. Followingly, we further investigated the correlation between MYL5 expression and immune-infiltrating cells in the tumor microenvironment, and results showed that MYL5 expression significantly correlated with immune-infiltrating cells and their gene markers in breast cancer patients. Furthermore, we integrated the information on MYL9-binding components and MYL9 expression-related genes in breast cancer for a series of enrichment analyses. However, we just used public databases of Oncomine, Kaplan–Meier plotter, GEO, PrognoScan, TCGA, TISIDB, and LinkedOmics datasets to demonstrate the effects of MYL5 on prognosis and immune infiltration in breast tumors. Additionally, we still need more evidence from cell and animal levels in detail.

## 5. Conclusion

In brief, we concluded that there is a possible prognostic molecular marker for good survival correlated with immune cell infiltration in breast cancer, called MYL5 expression. The low-expression level of MYL5 leads to the worsening of clinical features (primary tumor scope, lymphatic metastasis, and pathological stage of tumor and prognosis). This study firstly offers a relatively comprehensive understanding of the oncogenic roles of MYL5 for breast cancer.

## Figures and Tables

**Table 1:** Correlation between different clinicopathological factors and the expression of MYL5 gene in BRCA from TCGA database.

MYL5	Low expression			High expression	*p*	Statistic	Method
*n*		541	542				
T stage, *n* (%)					<0.001	20.59	Chi-squared.test
T1		114 (10.6%)	163 (15.1%)				
T2		347 (32.1%)	282 (26.1%)				
T3		58 (5.4%)	81 (7.5%)				
T4		21 (1.9%)	14 (1.3%)				

N stage, *n* (%)					0.083	6.68	Chi-squared.test
N0		260 (24.4%)	254 (23.9%)				
N1		167 (15.7%)	191 (18%)				
N2		69 (6.5%)	47 (4.4%)				
N3		34 (3.2%)	42 (3.9%)				

M stage, *n* (%)					1.000	0	Chi-squared.test
M0		478 (51.8%)	424 (46%)				
M1		11 (1.2%)	9 (1%)				

Pathologic stage, *n* (%)					0.040	8.33	Chi-squared.test
Stage I		73 (6.9%)	108 (10.2%)				
Stage II		324 (30.6%)	295 (27.8%)				
Stage III		121 (11.4%)	121 (11.4%)				
Stage IV		10 (0.9%)	8 (0.8%)				

Age, *n* (%)					0.168	1.9	Chi-squared.test
≤60		312 (28.8%)	289 (26.7%)				
>60		229 (21.1%)	253 (23.4%)				

Histological type, *n* (%)					<0.001	68.13	Chi-squared.test
Infiltrating ductal carcinoma		441 (45.1%)	331 (33.9%)				
Infiltrating lobular carcinoma		50 (5.1%)	155 (15.9%)				

Race, *n* (%)					0.007	9.94	Chi-squared.test
Asian		33 (3.3%)	27 (2.7%)				
Black or African American		71 (7.1%)	110 (11.1%)				
White		390 (39.2%)	363 (36.5%)				

PR status, *n* (%)					<0.001		Fisher.test
Negative		245 (23.7%)	97 (9.4%)				
Indeterminate		3 (0.3%)	1 (0.1%)				
Positive		271 (26.2%)	417 (40.3%)				

ER status, *n* (%)					<0.001		Fisher.test
Negative		193 (18.6%)	47 (4.5%)				
Indeterminate		2 (0.2%)	0 (0%)				
Positive		324 (31.3%)	469 (45.3%)				

HER2 status, *n* (%)					<0.001	14.1	Chi-squared.test
Negative		279 (38.4%)	279 (38.4%)				
Indeterminate		6 (0.8%)	6 (0.8%)				
Positive		105 (14.4%)	52 (7.2%)				

Molecular subtype, *n* (%)					<0.001	182.12	Chi-squared.test
Normal		22 (2%)	18 (1.7%)				
LumA		187 (17.3%)	375 (34.6%)				
LumB		103 (9.5%)	101 (9.3%)				
Her2		65 (6%)	17 (1.6%)				
Basal		164 (15.1%)	31 (2.9%)				

Menopause status, *n* (%)					0.286	2.5	Chi-squared.test
Pre		115 (11.8%)	114 (11.7%)				
Peri		25 (2.6%)	15 (1.5%)				
Post		349 (35.9%)	354 (36.4%)				

Anatomic neoplasm subdivisions, *n* (%)					0.522	0.41	Chi-squared.test
Left		287 (26.5%)	276 (25.5%)				
Right		254 (23.5%)	266 (24.6%)				

Radiation therapy, *n* (%)					0.521	0.41	Chi-squared.test
No		206 (20.9%)	228 (23.1%)				
Yes		275 (27.9%)	278 (28.2%)				

**Table 2:** Kaplan–Meier plotter to determine the effect of different clinicopathological factors on the expression of MYL5 gene and clinical prognosis in breast cancer.

Clinicopathological characteristics	Overall survival (*n* = 1879)			Relapse free survival (*n* = 4929)		
	*N*	Hazard ratio	*p*-value	*N*	Hazard ratio	*p*-value
ER status						
Positive	1309	0.83 (0.65–1.06)	0.13	3768	0.69 (0.61–0.78)	^*∗∗∗∗*^
Negative	570	0.62 (0.43–0.88)	^*∗∗*^	1161	0.73 (0.6–0.89)	^*∗∗*^
PR status						
Positive	156	1.5 (0.72–3.16)	0.28	926	0.76 (0.56–1.02)	0.07
Negative	291	0.65 (0.4–1.07)	0.088	925	0.72 (0.54–0.95)	^*∗*^
HER2 status						
Positive	420	0.78 (0.54–1.13)	0.19	882	0.71 (0.56–0.89)	^*∗∗*^
Negative	1459	0.65 (0.52–0.81)	^*∗∗∗*^	4047	0.58 (0.51–0.65)	^*∗∗∗∗*^
Intrinsic subtype						
Basal	404	0.65 (0.44–0.97)	^*∗*^	846	0.77 (0.62–0.97)	^*∗*^
Luminal A	794	1.29 (0.93–1.78)	0.12	2277	0.66 (0.56–0.78)	^*∗∗∗∗*^
Luminal B	515	0.73 (0.5–1.07)	0.11	1491	1.14 (0.94–1.38)	0.19
HER2^+^	166	0.43 (0.22–0.84)	^*∗*^	315	0.67 (0.45–1)	^*∗*^
Lymph node status						
Positive	452	0.75 (0.54–1.04)	0.086	1656	0.61 (0.52–0.73)	^*∗∗∗∗*^
Negative	726	0.53 (0.37–0.76)	^*∗∗∗*^	2368	0.72 (0.61–0.84)	^*∗∗∗∗*^
Grade						
1	175	0.49 (0.21–1.18)	0.1	397	0.54 (0.32–0.9)	^*∗*^
2	443	0.84 (0.56–1.27)	0.41	1177	0.77 (0.62–0.96)	^*∗*^
3	586	0.74 (0.55–1)	0.05	1300	0.75 (0.62–0.9)	^*∗∗*^
TP53 status						
Mutated	130	1.83 (0.93–3.63)	0.077	188	1.31 (0.78–2.21)	0.3
Wild type	197	0.33 (0.18–0.62)	^*∗∗∗*^	273	0.51 (0.32–0.81)	^*∗∗*^

OS: overall survival, RFS: relapse free survival, BRCA, and breast invasive carcinoma. ^*∗*^*p* < 0.05, ^*∗∗*^*p* < 0.01, ^*∗∗∗*^*p* < 0.001, and ^*∗∗∗∗*^*p* < 0.0001.

**Table 3:** Correlation analysis between MYL5 and relate genes and markers of B cell, macrophages, TAM, monocyte, and T cell exhaustion in GEPIA2.

Description	Gene markers	BRCA			
		Cancer		Normal	
		Cor	*p*	Cor	*p*
B cell	CD20 (KRT20)	−0.14	^*∗∗∗∗*^	0.051	0.59
	CD19	−0.061	^*∗*^	0.054	0.57
	CD38	−0.29	^*∗∗∗∗*^	−0.099	0.3

M1 Macrophage	NOS2	−0.15	^*∗∗∗∗*^	0.23	^*∗*^
	COX (PTGS2)	−0.2	^*∗∗∗∗*^	−0.17	0.075

M2 Macrophage	CD163	−0.21	^*∗∗∗∗*^	−0.056	0.56
	MS4A4A	−0.2	^*∗∗∗∗*^	−0.16	0.1
	VSIG4	−0.093	^*∗∗*^	−0.12	0.22

TAM	CCL2	−0.18	^*∗∗∗∗*^	−0.2	^*∗*^
	CD68	−0.22	^*∗∗∗∗*^	−0.21	^*∗*^
	IL10	−0.19	^*∗∗∗∗*^	−0.087	0.36

Monocyte	CD86	−0.22	^*∗∗∗∗*^	−0.28	^*∗∗*^
	CD16 (FCGR3A)	−0.19	^*∗∗∗∗*^	−0.22	^*∗*^

T cell exhaustion	PD1 (PDCD1)	−0.089	^*∗∗*^	0.024	0.8
	PD-L1 (CD274)	−0.16	^*∗∗∗∗*^	−0.36	^*∗∗∗∗*^
	CTLA4	−0.22	^*∗∗∗∗*^	−0.043	0.66

BRCA: breast invasive carcinoma, TAM: tumor-associated macrophage, None: correlation without adjustment, and C or, *R* value of Spearman's correlation. ^*∗*^*p* < 0.05, ^*∗∗*^*p* < 0.01, ^*∗∗∗*^*p* < 0.001, and ^*∗∗∗∗*^*p* < 0.0001.

## Data Availability

The datasets generated and analyzed during the current study are available in TCGA, Oncomine, GTEx, GEPIA 2, TIMER, TIMER2.0, PrognoScan, Kaplan–Meier Plotter, TISIDB, and LinkedOmics datasets.

## Abbreviations

MLC: Myosin light chain
MYL5: Myosin light chain 5
BRCA: Breast invasive carcinoma
OS: Overall survival
DMFS: Distant metastases-free survival
RFS: Relapse-free survival
PPS: Postprogression survival
PFS: Progression-free survival
TME: Tumor microenvironment
TAMs: Tumor-associated macrophages.
